# Crucial role of hsa-mir-503, hsa-mir-1247, and their validation in prostate cancer

**DOI:** 10.18632/aging.205213

**Published:** 2023-11-16

**Authors:** Ping Hu, Tao Wang, Hui Yan, Ying Huang, Yanjiao Zhao, Yuanyuan Gao

**Affiliations:** 1 The First Department of Medical Oncology, General Hospital of Ningxia Medical University, Yinchuan 750004, Ningxia, P.R. China; 2 The Second Department of Surgical Oncology, General Hospital of Ningxia Medical University, Yinchuan 750004, Ningxia, P.R. China; 3 The Second Department of Medicine Oncology, General Hospital of Ningxia Medical University, Yinchuan 750004, Ningxia, P.R. China; 4 The Third Department of Medicine Oncology, General Hospital of Ningxia Medical University, Yinchuan 750004, Ningxia, P.R. China

**Keywords:** prostate cancer, prognosis, microRNA, migration, invasion

## Abstract

Background: Prostate cancer (PC) is a common urinary system malignancy, and advanced PC patients had a poor prognosis due to recurrence or distant metastasis. Therefore, it’s imperative to reveal more details in tumorigenesis and prognosis of PC patients.

Methods: The miRNA and mRNA expression profile data of 485 PC patients were obtained from The Cancer Genome Atlas database. The univariate Cox regression was applied to screen miRNAs relating to prognosis of PC. Then miRTarBase was used to predict target mRNAs of miRNAs. The hsa-mir-503/hsa-mir-1247 knockdown in 22RV1 cells was established to evaluate the effect of these two miRNAs on tumor cell migration and invasion ability. Flow cytometry was used to detect the effect of hsa-mir-503/hsa-mir-1247 knockdown on 22RV1 apoptosis rate.

Results: Univariate Cox regression analysis identified hsa-mir-503 as a poor and hsa-mir-1247 as a favorable prognostic marker. Totally 649 target mRNAs were screened, among which DUSP19, FGF2, and SLC2A5 had a negative correlation with hsa-mir-503, while FGF2 and VSTM4 had a positive correlation with hsa-mir-1247. In 22RV1 cells, hsa-mir-503 was up-regulated, and hsa-mir-1247 was down-regulated. hsa-mir-503 knockdown attenuated the migration and invasion of 22RV1 cells, while hsa-mir-1247 knockdown exhibited the opposite effect. In addition, hsa-mir-503 knockdown promoted 22RV1 cell apoptosis. hsa-mir-1247 overexpression significantly inhibited the tumor growth of PC *in vivo*.

Conclusions: Herein, we demonstrated that hsa-mir-503 and hsa-mir-1247 could serve as new prognostic markers of PC, and hsa-mir-1247 had great potential to inhibit PC progression by suppressing the migration and invasion ability *in vitro* and *in vivo*.

## INTRODUCTION

Prostate cancer (PC) is one of the most common urinary system malignancies, ranking second among male malignant tumors, and it has caused a heavy burden on male health [1]. In the worldwide, the incidence rate of standardized age of PC increased from 30.5 cases per 100 thousand population in 1990 to 37.9 cases per 100 thousand population in 2017, showing a gradual increasing trend [2]. The great development and popularity of prostate-specific antigen (PSA) screening and prostate needle biopsy technology makes it possible for more patients with PC to be diagnosed at an early-stage [3]. Although most of these patients could benefit from the radical prostatectomy, unfortunately, approximately 20–40% localized PC patients would eventually suffer from a biochemical recurrence [4]. Nevertheless, the treatment strategy of PC has made significant progress and the survival rate of patients has been significantly improved [5], the overall survival rate (OSR) of patients has not been significantly improved, and the 5-year OSR was only about 28% [6]. Accurate prognosis stratification provides the opportunity to enhance patient survival chances [7]. Therefore, new prognostic markers are urgently needed to redefine the prognosis of PC patients.

The microRNAs (miRNAs) are noncoding RNAs of ~22 nucleotides, which affect multiple biological pathways by regulating the expression of target genes [8]. In multiple stages of tumor occurrence, development and deterioration, miRNAs have widely participated in tumor cell proliferation, angiogenesis, invasion and migration, thus affecting tumor progression [9, 10]. Recent studies have revealed the role of miRNAs in PC. For instance, one report has already showed that miR-139 could inhibit the growth and migration of PC cells by cell cycle arrest [11]. Another study has revealed that miR-215-5p is lowly expressed in PC samples, and miR-215-5p is able to reduce PC metastasis by down-regulating PGK1 [12]. Meanwhile, several studies have focused on the prognostic value of miRNAs in PC. For example, Bian et al. have established a prognostic model for PC patients based on 15 related miRNAs [13]. Bi et al. showed that PC patients with high expression of miR-153 showed a lower 5-year OSR, which suggested miR-153 might be a prognostic marker of PC patients. These studies inspire us to further explore crucial miRNAs with great prognostic value and clinical target potential in PC.

Herein, the purpose of our present work is to explore promising hub miRNAs as well as their targets in PC patients, employing bioinformatics analysis and wet experiments. The findings of our study are expected to give deepening insights into the possible pathogenic and prognostic factors of PC, which would be indirectly conducive to the better clinical management strategy of PC.

## RESULTS

### hsa-mir-503 and hsa-mir-1247 were hub miRNAs relating to PC patients’ DFS

**Figure 1:**
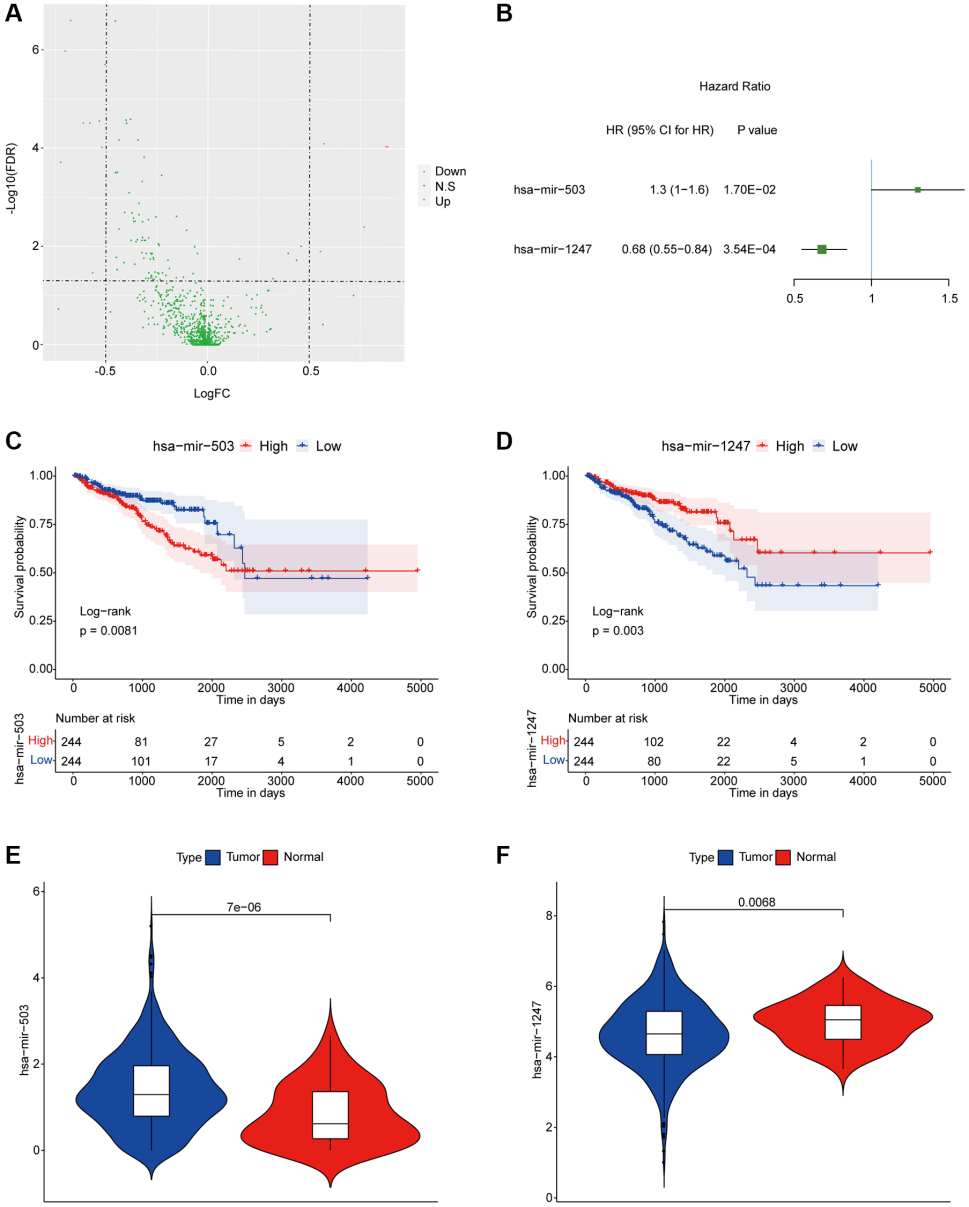
**hsa-mir-503 and hsa-mir-1247 were associated with prognosis of PC.** (**A**) Volcanic map of differential expression of miRNAs. The blue dots were down-regulated miRNAs, and the red dots were up-regulated miRNAs. (**B**) Univariate analysis of two miRNAs significantly associated with DFS. Abbreviation: HR: hazard ratio. 95% CI: 95% confidence interval. (**C**, **D**) KM survival curve. *p* value was obtained based on log-rank test. (**E**, **F**) Violin diagram of differential expression levels of two miRNAs in cancer and para-cancer samples. *P* value was calculated by Wilcoxon method.

We previously divided PC patients into three groups with significant differences in disease free survival (DFS) according to proportions of tumor-infiltrating immune cell subpopulations, in which PC samples in cluster 1 had better DFS while PC samples in cluster 3 had worse DFS [14]. In present study, we further screened miRNAs associated with DFS of PC patients. Firstly, we analyzed all differentially expressed miRNAs (DEMs) of PC samples from the poor DFS group (cluster 3) and the better DFS group (cluster 1), and the detailed sample list of cluster 1/cluster 3 was included in Supplementary Table 1. As the results, there were 14 DEMs between two groups, including 9 down-regulated and 5 up-regulated miRNAs (Figure 1A). Among them, the hsa-mir-503 and hsa-mir-1247 were significantly correlated with DFS by univariate Cox regression analysis (Figure 1B). Then, basing on the median expression values of hsa-mir-503 (median expression level, 1.284408119) and hsa-mir-1247 (median expression level, 4.639203101), all PC patients were divided in high and low expression groups, respectively. And hsa-mir-503 was a risk factor and the higher the expression, the worse the prognosis (HR (hazard ratio) = 1.3, 95% CI (confidence interval): 1–1.6, *p* = 0.017); while hsa-mir-1247 was a protective factor, the higher the expression, the better the prognosis (HR = 0.68, 95% CI: 0.55–0.84, *p* = 0.000354) (Figure 1C, 1D). As a member of miR-15/16 family, miR-503 was down-regulated in several tumors, such as oral cancer and gastric cancer, while it was overexpressed in retinoblastoma and adrenocortical carcinoma, indicating that the expression pattern of miR-503 was tissue or disease-specific [15]. In this study, we found that the expression level of hsa-mir-503 in PC samples was significantly higher than that in para-cancer samples (Figure 1E, *p* = 7E-06), the expression of hsa-mir-1247 was down-regulated in the tumor sample (Figure 1F, *p* = 0.0068).

### hub miRNAs’ targets and their functions in PC patients

**Figure 2:**
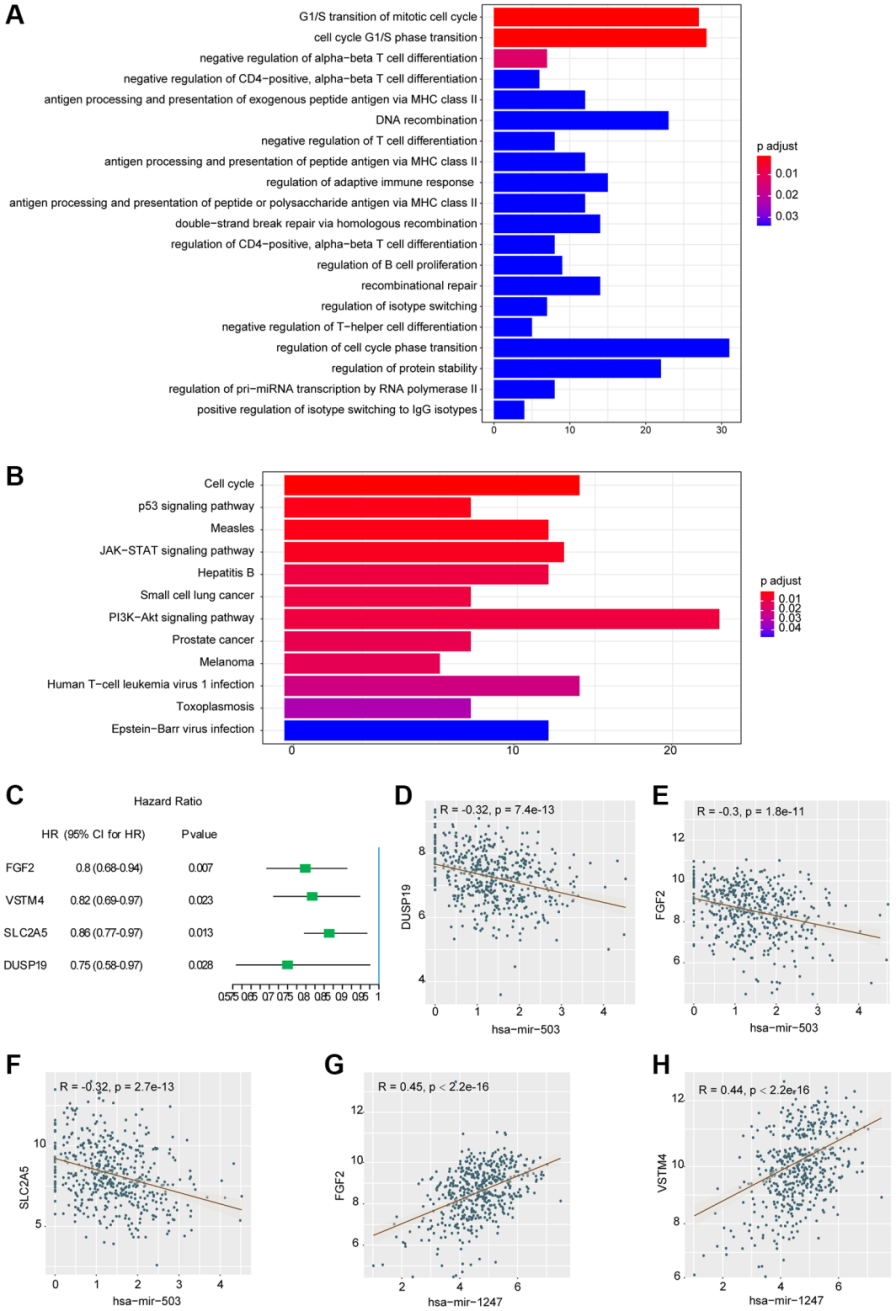
**Results of functional enrichment analysis and correlation analysis.** (**A**) Histogram of the top 20 GO terms with the most significant enrichment (the horizontal axis was the number of genes, and the vertical axis was the name of GO terms). (**B**) Histogram of the 12 KEGG pathways with the most significant enrichment (the horizontal axis represented the number of genes, and the vertical axis represented the name of KEGG pathway). (**C**) Univariate analysis of three mRNAs significantly associated with DFS. Abbreviation: HR: hazard ratio. 95% CI: 95% confidence interval. (**D**–**H**) Correlation scatter with fitting line.

In this study, miRTarBase database was used to predict the target mRNAs of hsa-mir-503 and hsa-mir-1247, and a total of 649 target mRNAs were identified. These miRNAs potentially regulated their target mRNAs to influence the tumorigenesis and progression of PC. Subsequently, functional and pathway enrichment analysis were performed for the target mRNAs. The GO-biological process revealed that these mRNAs were enriched in regulation of cell cycle phase transition, DNA recombination, DNA metabolic process, protein stability, immune cell proliferation and differentiation, protein kinase complex, serine/threonine kinase activity, ubiquitin protein ligase binding, etc. (Figure 2A, Supplementary Table 2). KEGG analysis showed that these target mRNAs were enriched in cell cycle, p53 signaling pathway, PI3K-Akt signaling pathway, JAK-STAT signaling pathway, etc., (Figure 2B, Supplementary Table 2). Most of these pathways have been shown to be involved in tumor invasion, metastasis and drug resistance [16, 17].

### Correlation between hsa-mir-503/hsa-mir-1247 and their target mRNAs in PC patients

To further investigate the relationship between hsa-mir-503/hsa-mir-1247 and 649 target mRNAs, WGCNA package in R programming software was used for correlation analysis. A total of 868 miRNA-mRNA correlation relationships were screened, including 339 pairs of negative correlation (NeC) and 529 pairs of positive correlation (PoC) (*p* < 0.05, the results were listed in Supplementary Table 3). We selected the top 10 mRNA from the PoC and NeC pairs and further evaluated their prognostic value by univariate Cox regression analysis. The hazard ratio value showed that 4 mRNAs were significantly correlated with DFS (*p* < 0.05, Figure 2C), including FGF2 (fibroblast growth factor 2), VSTM4 (V-set and transmembrane domain containing 4), SLC2A5 (solute carrier family 2 member 5), and DUSP19 (dual specificity phosphatase 19). And the higher the expression level, the better the prognosis. Among the 4 mRNAs, DUSP19, FGF2, and SLC2A5 were negatively correlated with hsa-mir-503 (Figure 2D–2F), while FGF2 and VSTM4 were positively correlated with hsa-mir-1247 (Figure 2G, 2H).

### The expressions of crucial target mRNAs in PC and para-cancer samples

**Figure 3:**
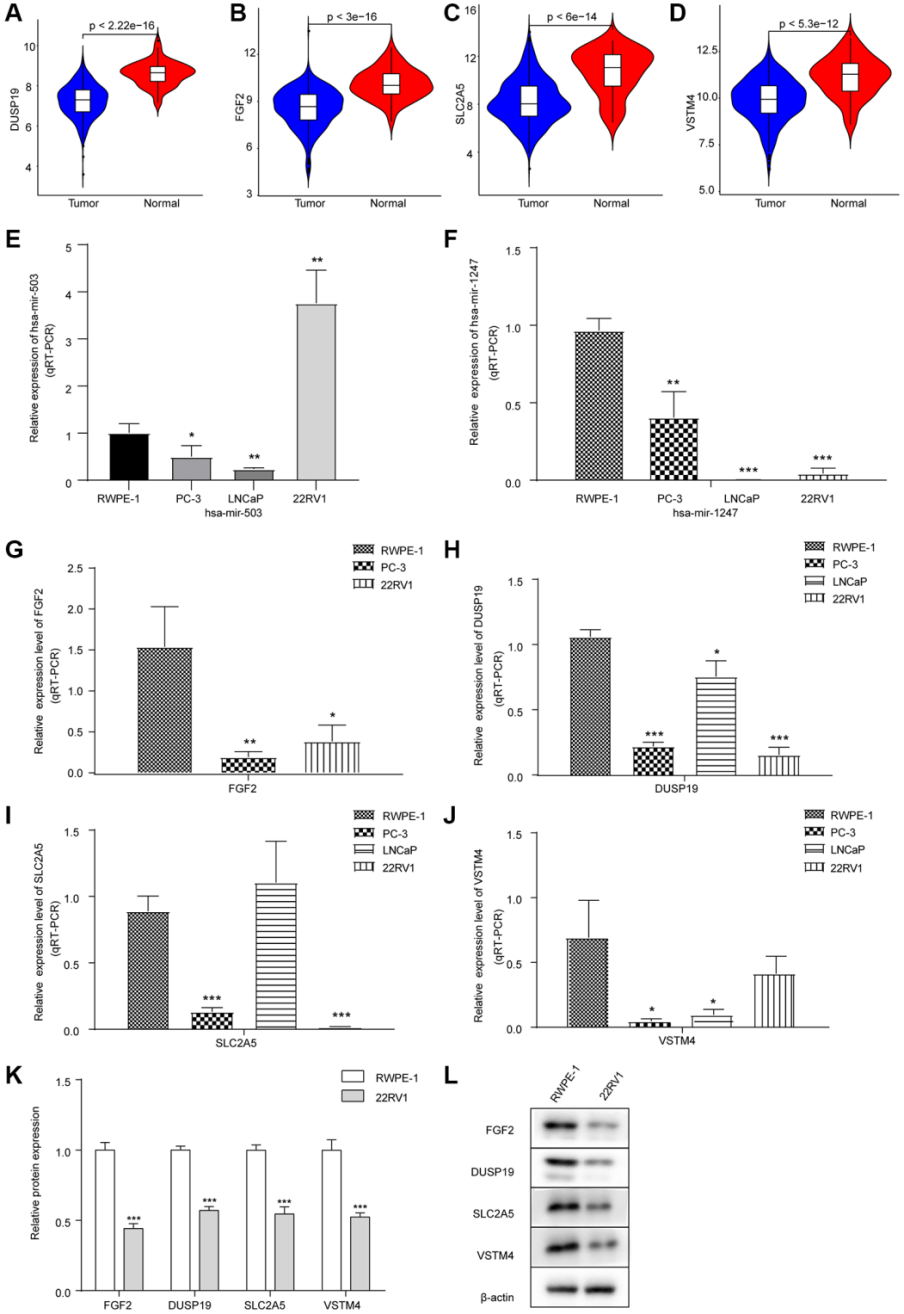
**Expression levels of hsa-mir-503/hsa-mir-1247 and their target mRNAs in tumor samples and three PC cell lines.** (**A**–**D**) Violin diagram of expression differences of DUSP19, FGF2, SLC2A5 and VSTM4 between cancer and para-cancer samples. The horizontal axis was the sample group, the vertical axis was the expression level, and *p* value was calculated by Wilcoxon method. (**E**) Expression of hsa-mir-503 in PC-3, LNCaP and 22RV1 cells. (**F**) Expression of hsa-mir-1247 in PC-3, LNCaP and 22RV1 cells. (**G**–**J**) Expression of FGF2, DUSP19, SLC2A5 and VSTM4 in PC-3, LNCaP and 22RV1 cells. (**K**, **L**) The expressions of FGF2, DUSP19, SLC2A5 and VSTM4 in 22RV1 cells were detected by WB (^*^p < 0.05, ^**^p < 0.01, ^***^p < 0.001 vs. RWPE-1).

As shown in Figure 3A–3D, the expression levels of these 4 mRNAs in PC samples were significantly lower than that in para-cancer samples. Meanwhile, we found that FGF2 was regulated by hsa-mir-503 and hsa-mir-1247 via the correlation analysis and miRTarBase database prediction. Previous studies have shown that mir-503 down-regulated expression of FGF2 by directly targeting its 3’-UTR [18]. Whether such a mechanism existed in PC needs to be verified by further in-depth and detailed experimental data. Ultimately, low expression of FGF2, VSTM4, SLC2A5, and DUSP19 predicted poor DFS results.

### Expression levels of hsa-mir-503/hsa-mir-1247 and their target mRNAs in PC cell lines

Next, the expression of hsa-mir-503 and hsa-mir-1247 and their target mRNA in three PC cell lines was verified by qRT-PCR. Compared with RWPE-1 cells, both hsa-mir-503 and hsa-mir-1247 were down-regulated in PC cells, except that hsa-mir-503 was up-regulated in 22RV1 cells (Figure 3E, 3F). Moreover, compared with RWPE-1 cells, in PC-3 cells, the levels of FGF2, DUSP19, SLC2A5 and VSTM4 were decreased; in LNCaP cells, the levels of DUSP19 and VSTM4 were decreased, while the difference in SLC2A5 level was not significant, and FGF2 was not detected; in 22RV1 cells, the levels of FGF2, DUSP19 and SLC2A5 were decreased, while the difference in VSTM4 level was not significant (Figure 3G–3J). In general, the expression levels of four genes were consistent with the results of bioinformatics analysis.

The hsa-mir-503 was highly expressed in 22RV1 cells, which triggered us to explore the effect of hsa-mir-503 and hsa-mir-1247 expression on PC progression. We further performed WB on RWPE-1 and 22RV1, and the results showed that compared with RWPE-1 cells, the expression levels of FGF2, DUSP19, SLC2A5 and VSTM4 in 22RV1 cells were significantly decreased (Figure 3K, 3L), which suggested that 22RV1 was a suitable PC cell type for this study.

### Down-regulation of hsa-mir-503/hsa-mir-1247 affected the expression of their target mRNAs in PC

**Figure 4:**
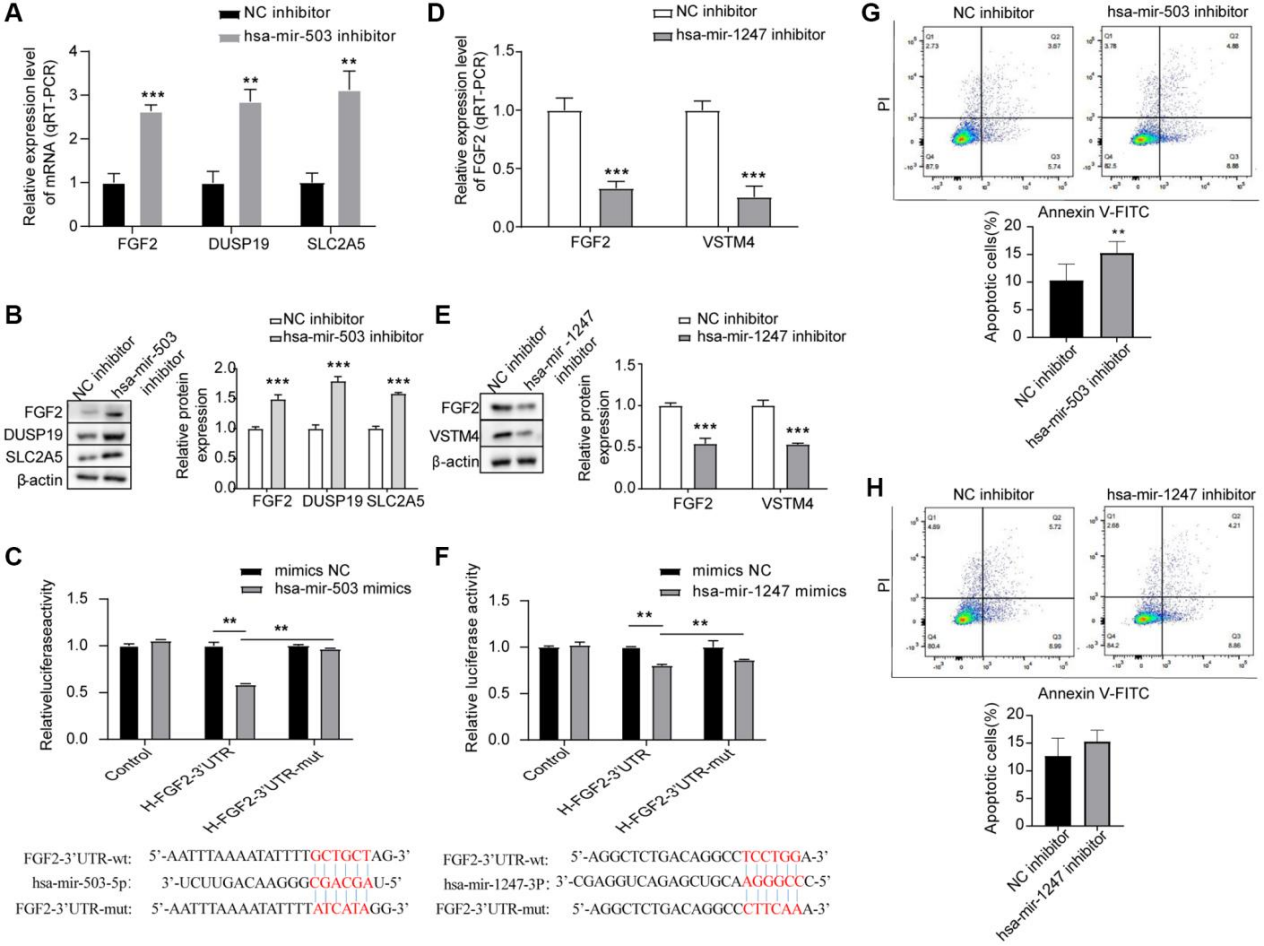
**Effects of hsa-mir-503/hsa-mir-1247 knockdown on target mRNAs expression and 22RV1 cells apoptosis.** (**A**, **B**) The mRNA and protein levels of FGF2, DUSP19, and SLC2A5 were significantly increased after hsa-mir-503 knockdown. ^*^p < 0.05, ^**^p < 0.01. (**C**) The binding of hsa-mir-503 to FGF2-3’-UTR verified by dual luciferase reporter assay (^**^p < 0.01). (**D**, **E**) The mRNA and protein levels of FGF2 and VSTM4 were significantly decreased after hsa-mir-1247 knockdown. (**F**) The binding of hsa-mir-1247 to FGF2-3’-UTR verified by dual luciferase reporter assay (^**^p < 0.01). (**G**) The apoptotic rate of 22RV1 cells was significantly increased after hsa-mir-503 knockdown. (**H**) The apoptotic rate of 22RV1 cells was not significantly changed after hsa-mir-503 knockdown (^*^p < 0.05, ^**^p < 0.01, ^***^p < 0.001 vs. NC inhibitor).

To further verify whether hsa-mir-503 and hsa-mir-1247 have regulatory effects on FGF2, DUSP19, SLC2A5 and VSTM4, we interfered with the expression of these two miRNAs in 22RV1 cells by hsa-mir-503 inhibitor and hsa-mir-1247 inhibitor. As shown in Figure 4A, 4B, the mRNA and protein expression levels of FGF2, DUSP19 and SLC2A5 were significantly increased when hsa-mir-503 was knocked down. These results confirmed that hsa-mir-503 could negatively regulate the expression of FGF2, DUSP19 and SLC2A5. Moreover, the predicted binding sites between FGF2-3′-UTR and hsa-mir-503, hsa-mir-1247 were displayed in Figure 4C, 4F, respectively. After transfection of hsa-mir-503 mimic, luciferase activity of FGF2-3′-UTR declined significantly, but luciferase activity of FGF2-3′-UTR-mut was unaffected (Figure 4C). These results indicated that mutation locus of FGF2-3′-UTR was probably a crucial binding site of hsa-mir-503. By contrast, the mRNA and protein expression levels of FGF2 and VSTM4 were significantly decreased after hsa-mir-1247 was knocked down (Figure 4D, 4E). Meanwhile, our dual luciferase data indicated a significant decline of luciferase activity of FGF2-3′-UTR, while luciferase activity of FGF2-3′-UTR-mut was unaffected after transfection of hsa-mir-1247 mimic (Figure 4F), demonstrating the potential binding of hsa-mir-1247 to FGF2-3′-UTR.

### Effects of hsa-mir-503 and hsa-mir-1247 on the apoptosis of PC cells

Apoptosis rates of the 22RV1 cells were estimated by flow cytometry after the interference to hsa-mir-503 and hsa-mir-1247, respectively. After transfection of has-mir-503 inhibitor and NC inhibitor in 22RV1 cells for 24 h, cells were collected for apoptosis detection according to the operation procedure. The results showed that has-mir-503 inhibitor group promoted cell apoptosis compared with NC inhibitor group (Figure 4G). However, knockdown of hsa-mir-1247 expression had no significant effect on the apoptosis rate of 22RV1 cells (Figure 4H).

### Effects of hsa-mir-503/hsa-mir-1247 knockdown on migration and invasion ability of 22RV1 cells

**Figure 5:**
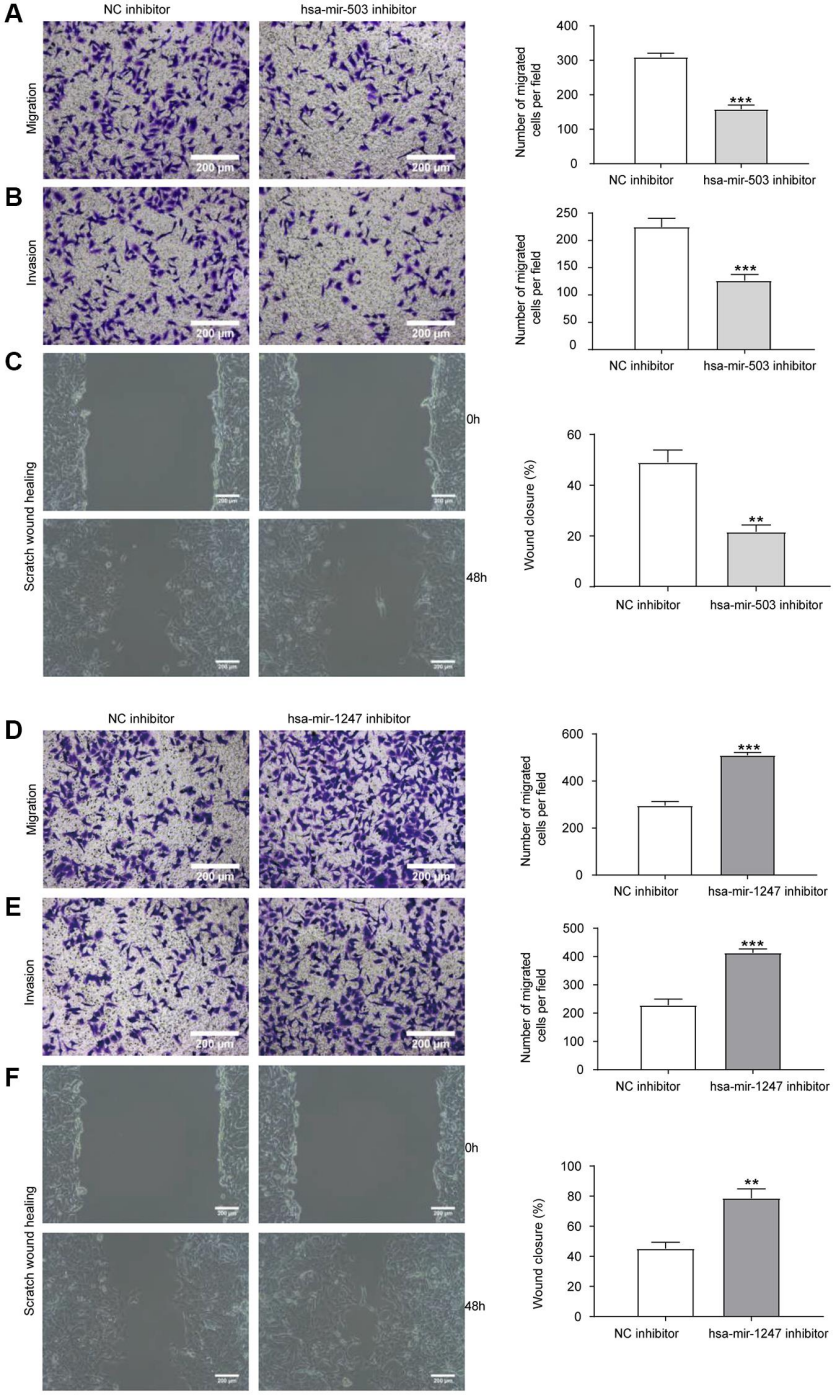
**Effects of hsa-mir-503/hsa-mir-1247 knockdown on migration and invasion ability of 22RV1 cells.** (**A**) The migratory ability of 22RV1 cells was significantly suppressed after hsa-mir-503 knockdown. (**B**) The invasive ability of 22RV1 cells was significantly suppressed after hsa-mir-503 knockdown. (**C**) Knockdown of hsa-mir-503 inhibited the lateral migration ability of 22RV1 cells. (**D**) The migratory ability of 22RV1 cells was significantly increased after hsa-mir-1247 knockdown. (**E**) The invasive ability of 22RV1 cells was significantly increased after hsa-mir-1247 knockdown. (**F**) Knockdown of hsa-mir-1247 promoted the lateral migration ability of 22RV1 cells (^*^p < 0.05, ^**^p < 0.01, ^***^p < 0.001 vs. NC inhibitor).

Migration and invasion were necessary characteristics for malignant progression [19]. Transwell chamber was used for cell migration and invasion analysis. As shown in Figure 5A, 5B, compared with NC inhibitor group, knockdown of hsa-mir-503 expression in 22RV1 resulted in a significant reduction of cells migrating to the bottom surface of the Transwell chamber. The scratch wound healing experiment further demonstrated that knockdown of hsa-mir-503 reduced the migration and invasion ability of 22RV1 cells (Figure 5C). Interestingly, knockdown of hsa-mir-1247 enhanced the migration and invasion of 22RV1 cells (Figure 5D–5F). This evidence suggested that hsa-mir-503 and hsa-mir-1247 might have opposite effects in the progression of PC, and hsa-mir-503 has the potential to promote tumor metastasis, whereas hsa-mir-1247 exhibited great potential as an anti-tumor factor in PC.

### hsa-mir-1247 transfection significantly inhibited the tumor growth of PC *in vivo*

**Figure 6:**
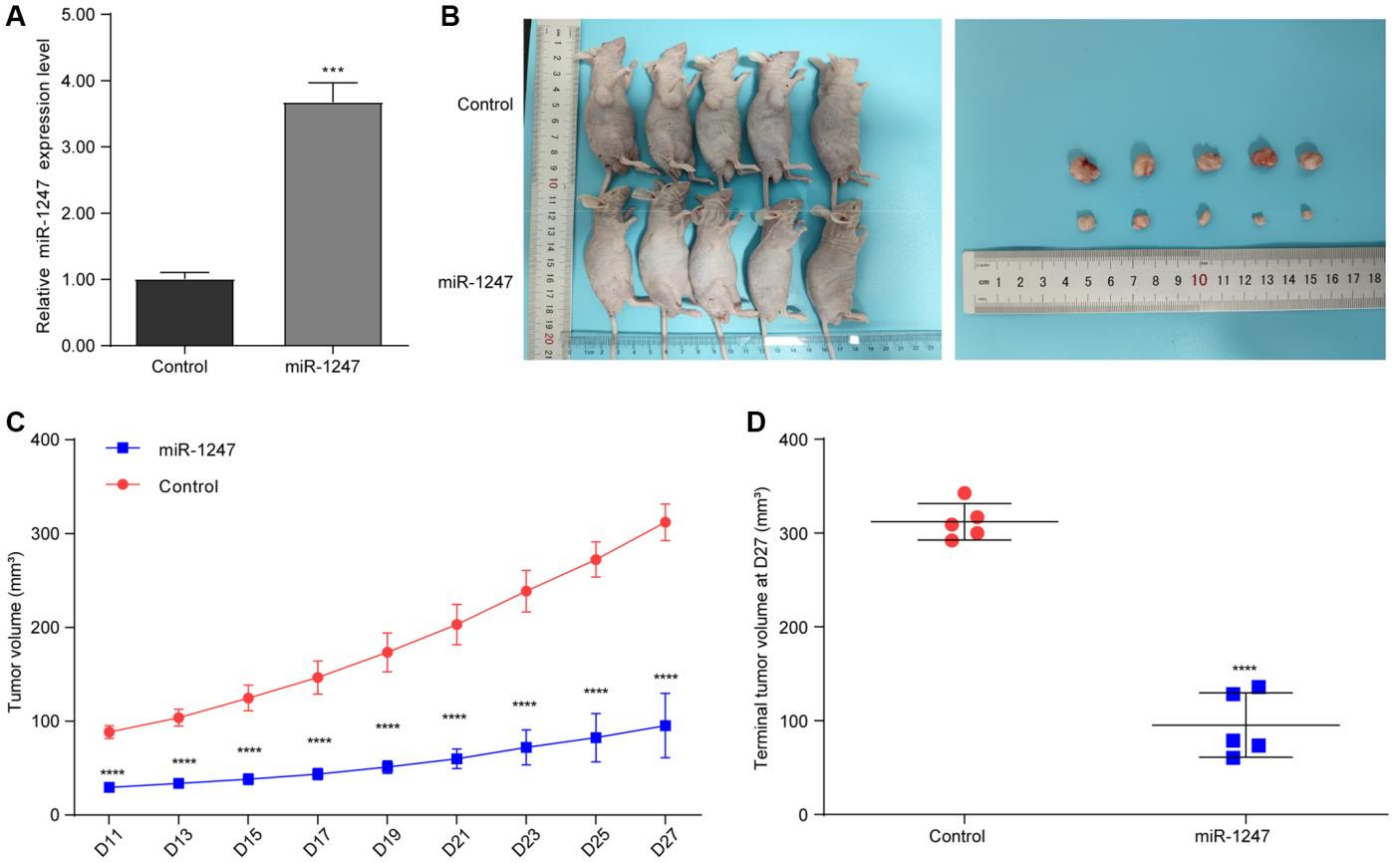
**hsa-mir-1247 transfection significantly inhibited the tumor growth of PC *in vivo*.** (**A**) The qPCR was used to validate the overexpression of hsa-mir-1247 in 22RV1 cells. (**B**) Photographs of animals (upper panel) and xenograft tumors (lower panel). (**C**) Tumor volumes. (**D**) Terminal tumor volumes. ^***^p < 0.001; ^****^p < 0.0001.

Considering the crucial tumor growth suppressing potential of hsa-mir-1247, we have further explored its role in a murine xenograft mouse model of 22RV1 cells. We have successfully established a stable 22RV1 cell line overexpressing hsa-mir-1247 (Figure 6A). Then, after xenograft mouse model construction, the tumor volumes of hsa-mir-1247-transfected group were significantly smaller than control group (Figure 6B). Moreover, tumor growth of hsa-mir-1247-transfected group was significantly inhibited, comparing to control group (Figure 6C, 6D). Our data indicated that hsa-mir-1247 overexpression significantly inhibited the tumor growth of PC *in vivo*.

## DISCUSSION

In recent years, the morbidity of PC in China has also shown a significant upward trend, and most patients were already in advanced stage when diagnosed [20]. Most patients with early PC could benefit from surgery or chemotherapy and acquired a good prognosis. However, it was frustrating that patients who suffered from advanced PC had a poor prognosis due to recurrence or distant metastasis [21]. Therefore, it is necessary to identify biomarkers that predict the prognosis of PC patients. More and more studies have shown that miRNAs could establish complex gene regulatory networks, affect tumorigenesis and progression through a variety of biological pathways, and have prognostic potential [22]. In present study, we screened and validated two miRNAs with prognostic value (hsa-mir-503 and hsa-mir-1247) and further investigated their effects on migration and invasion of 22RV1 cells.

Previous studies have demonstrated the prognostic potential of hsa-mir-1247 in breast cancer [23, 24], but the prognosis of hsa-mir-503 and hsa-mir-1247 in PC has not yet been reported. In our present study, hazard ratio indicated that hsa-mir-503 was a risk factor that negatively correlated with prognosis. On the contrary, hsa-mir-1247 was a protective factor that associated with a better prognosis. Highlighted, we also demonstrated that hsa-mir-503 was significantly overexpressed in PC cells, while hsa-mir-1247 was down-regulated. Meanwhile, their target mRNAs and the corresponding genes’ prognostic value were evaluated. Results show that four were protective factors, and the expression level was directly proportional to the prognosis. According to the results of go and KEGG analysis, we speculated that hsa-mir-503 and hsa-mir-1247 might affect the prognosis of PC from many aspects, such as cell cycle, p53 signaling pathway, JAK/STAT signaling pathway, PI3K/Akt signaling pathway and DNA recombination by regulating these 4 mRNAs. All these results proved the significance of hsa-mir-503 and hsa-mir-1247 in clinical prognosis and revealed how these miRNAs affected tumor progression.

Given the high expression of hsa-mir-503 and the low expression of hsa-mir-1247 in 22RV1, we were interested in their function in PC cells. Therefore, we knocked down the expression of hsa-mir-503 and hsa-mir-1247, and then used Transwell chamber to evaluate the ability of cell invasion and migration. As expected, knockdown of hsa-mir-503 decreased the migration and invasion ability of PC cells, while knockdown of hsa-mir-1247 had the opposite trend. These were consistent with previous studies [25, 26]. Moreover, we noticed that knockdown of has-mir-503 significantly increased the apoptosis rate of 22Rv1 cells, suggesting that has-mir-503 might be necessary for the survival of PC cells. These results suggested that hsa-mir-503 had a potential role in promoting tumor cell metastasis, and hsa-mir-1247 could inhibit tumor development, which may explain why hsa-mir-503 was analyzed as a risk factor and hsa-mir-1247 was analyzed as a protective factor mentioned above.

In addition, DUSP19, FGF2, and SLC2A5 were predicted to be the target mRNA of hsa-mir-503. DUSP19 was identified as a MAPK phosphatase and DUSP19 up-regulation decreased the expression of matrix metalloproteinases (MMPs) through inactivating JAK2/STAT3 pathway [27]. MMPs were known to degrade the extracellular matrix, which was critical for tumor invasion and metastasis [28]. In hsa-mir-503 knockdown cells, DUSP19 was highly expressed, and the cell invasion ability was weakened, which may be attributed to the high expression of DUSP19 inhibiting the expression of MMPs, resulting in a weakened ability of cells to degrade Matrigel. More recently, Chang et al. have revealed that DUSP19-mediated VEGFR3 dephosphorylation exerted a crucial role in PTEN’s regulation of the invasiveness of pancreatic neuroendocrine tumors [29], while whether DUSP19 could play similar role in PC has been rarely studied. Moreover, the correlation of FGF2 with tumor cell migration/ invasion has been explored in multiple tumors, such as cervical cancer [30] and lung cancer [31]. A recent study in pancreatic cancer, miRNA-203-3p has been indicated to suppress the invasion and migration of tumor cells via downregulating FGF2 [32]. However, some studies have reported that FGF2 was up-regulated in thyroid cancer [33] and SLC2A5 was up-regulated in lung adenocarcinoma [34], which was inconsistent with our results may be due to the different cancer type and genetic background. Actually, our present work mainly focused on the role of hsa-mir-503 and hsa-mir-1247 in PC employing bioinformatics tool, as well as *in vitro* and *in vivo* wet lab experiments. Nevertheless, there were still some interesting mechanism aspects that remains to be illustrated. For instance, whether hsa-mir-503 initiates cell migration through epithelial mesenchymal transformation was unknown, which would be the focus of our future work.

Overall, we screened and verified the role of hsa-mir-1247 and hsa-mir-503 in predicting the prognosis of PC patients. These two miRNAs were strongly associated with better and poorer prognosis, respectively, which demonstrated the ability to distinguish different prognosis. Currently, we are collecting more PC samples, and further designing experiments to elucidate the molecular mechanisms that they affect the progression of PC and validate our findings. Collectively, after further deepening investigation, our data involving hsa-mir-1247 and hsa-mir-503 in PC would provide valuable information for not only therapeutic target but also diagnostic markers for PC patients, which is conducive to a better management strategy for clinical PC patients.

## MATERIALS AND METHODS

### Study objects

The miRNA and mRNA expression profile data and clinical information of 485 PC patients with complete survival information were downloaded from The Cancer Genome Atlas database (TCGA, https://tcga-data.nci.nih.gov/tcga/). The detailed clinical information of 485 PC patients was shown in Table 1, and the entire sample information were included in Supplementary Table 4.

**Table 1:** Clinicopathological characteristics of PRAD patients from TCGA database.

**Characteristics**		**Patients (*N* = 485)**	
		**No.**	**%**
**Age**	≤61 (Median)	245	50.52%
	>61 (Median)	240	49.48%
**Race**	White	403	83.09%
	Black or African American	55	11.34%
	Asian	12	2.47%
	American Indian or Alaska native	1	0.21%
	Unknown	14	2.89%
**Metastasis**	Yes	16	3.29%
	No	469	96.71%
**Survival time**	Long (>5 years)	83	17.11%
	Short (<5 years)	402	82.89%
**OS status**	Dead	4	0.82%
	Alive	481	99.18%

### Analysis of differentially expressed miRNA and prediction of target mRNA

Based on the limma [35] package of R programming software (version 4.0.3), DEMs were screened according to the threshold of absolute value of differential expression multiple >0.5 and FDR ≤0.05. For the screened differential miRNAs, miRTarBase database was used to predict their target mRNAs [36].

### Functional and pathway enrichment analysis

Gene Ontology (GO) and Kyoto Encyclopedia of Genes and Genomes (KEGG) pathway enrichment analyses were performed using the “clusterProfiler” package of R programming software [37]. *p* < 0.05 was used to screen the significantly enriched GO term and KEGG pathway.

### Survival analysis

The survival and survminer packages of R programming software were used to estimate the OSR of prognostic patients based on Kaplan-Meier (KM) method, and the significance of OSR difference among groups was determined by log-rank test.

### Cell culture and transfection

Human normal prostate epithelial cells RWPE-1 and three PC cells (including: PC-3, LNCaP, and 22RV1 cells) were purchased from Nanjing Siri Biotechnology Co., Ltd., (Nanjing, China). All cell line authentications were conducted using short tandem repeat profiling and all cells were free of mycoplasma infection. The cells were cultured in RPMI1640 medium (31800022-10, Gibco, USA) supplemented with 1% penicillin/streptomycin and 10% fetal bovine serum (10270-106, Gibco) at 37°C in 5% CO_2_.

hsa-mir-503 inhibitors, hsa-mir-1247 inhibitors, and inhibitor controls (NC inhibitors) were purchased from RiboBio (Guangzhou, China). 22RV1 cells were inoculated in 6-well plate at a density of 4 × 10^5^ cells/well 24 hours before transfection to ensure 70% cell confluence at the moment of transfection. Transfection was conducted for 48 h using Lipofectamine 2000 (11668019, Thermo Fisher Scientific, USA).

### RNA extraction and quantitative reverse-transcription polymerase chain reaction (qRT-PCR)

Trizol kit (R1100, SolarBio, Beijing, China) was used to extract total RNA from cells, and ultraviolet spectrophotometer (SMA2000, Thermo Fisher Scientific) was used to detect the quantification and concentration of extracted RNA. After qualified detection, miRNA reverse transcription was performed using the Bulge Loop miRNA QRT-PCR Starter Kit (C10211-2, RiboBio, Guangzhou, China), and then qPCR was performed using the Bulge Loop miRNA QRT-PCR Primer (RiboBio) under the qPCR fluorescence quantitative system (FQD-96A, Hangzhou Bioer Technology Co., Ltd., Hangzhou, China) as follows: qPCR was performed at 95°C for 10 min for pre-denaturation. The 40 cycles were as follows: 95°C for 2 s, 60°C for 20 s, 70°C for 10 s. The reference gene was U6. mRNA reverse transcription was performed using reverse transcription kit (11123ES60, Yeasen Biotechnology Co., Ltd., Shanghai, China), and then qPCR was performed using qPCR kit (11123ES60, Yeasen) under qPCR fluorescence quantitative system. The procedure was as follows: 95°C, 3 min pre-denaturation. The 40 cycles were as follows: 95°C, 15 s, 60°C, 45 s. GAPDH was used for internal reference genes, and primer sequences were shown in Table 2, with 3 replicates per sample. mRNA expression level was calculated by 2^−ΔΔ^CT formula.

**Table 2:** Primer sequences for qRT-PCR.

**Genes**	**Forward primer (5′–3′)**	**Reverse primer (5′–3′)**	**Product (bp)**
DUSP19	CACCAGGGTGACAACGCTAA	CCCACTGCTCGGCTCTACTT	107
SLC2A5	CAGCAGAGTCGCCACATCAT	ACCCAAAGGCAGCTATCAGG	311
VSTM4	AAGCGGAAATCCAGAGTGAGA	TGACCAGCATGAAGAGCAGAA	65
FGF2	AAGAGCGACCCTCACATCAAG	CGTTTCAGTGCCACATACCAA	224
GAPDH	GGAGCGAGATCCCTCCAAA	GGCTGTTGTCATACTTCTCATGG	197

### Western blot analysis (WB)

The following antibodies were used for WB analysis: FGF2 (GTX84501, 1:5000, GeneTex, USA), DUSP19 (GTX104197, 1:1000, GeneTex, USA), SlC2A5 (GTX12098, 1:5000, GeneTex, USA), VSTM4 (ab252933, 1:1000, Abcam, UK), β-actin (23660-1-ap, 1:5000, Proteintech, USA), sheep-anti-rabbit IgG-HRP (BK0027, 1:2000, Best Biological Technology, China). Briefly, cells were lysed with RIPA (Best Biological Technology, China) to extract protein, and the protein concentration was determined by BCA method. The protein samples were further electrophoresed by SDS-PAGE and transferred to PVDF membrane (IPVH00010, Millipore, USA). The PVDF membrane was immersed in the blocking solution (5% skimmed milk), gently shaken for 2 h, then incubated with the primary antibody and then with the secondary antibody. Finally, the membrane was washed three times with TBST for 10 min each time, the membrane was developed with a chemiluminescence ECL detection kit (S17851, Yeasen). Image J software was used to analyze the gray value of the strip.

### Dual luciferase reporter assay

The 3′-UTR of FGF2, containing hsa-miR-503, hsa-miR-1247 binding sites and corresponding mutant sequences, was inserted into pmirGLO vectors, which was co-transfected with hsa-miR-503 mimic and hsa-miR-1247 mimic, respectively. Then the luciferase report analysis was conducted following the instructions of luciferase assay kit (E1910, Promega, USA). The luciferase activity was subsequently calculated with activity of firefly luciferase/activity of Renilla luciferase. There were three repeats in each group.

### Cell migration assay

Transwell migration assay was used for migration analysis. 22RV1 cells were inoculated with 4 × 10^4^ cells/well in the upper chamber of Transwell cell (3422, Thermo Fisher Scientific), and then the Transwell cell was placed into a 24-well plate (600 μL complete medium was added in the 24-well plate) and cultured in an incubator at 37°C with 5% CO_2_. After culturing for 24 h, the cells that migrated to the bottom surface were fixed with 4% paraformaldehyde for 10 min, and then stained with 0.5% crystal violet for 15 min. Five visual fields were randomly observed with a microscope, and then photographed, counted, and statistically analyzed.

### Cell invasion assay

Transwell chamber was also used for cell invasion assay. 22RV1 cells were inoculated with 4 × 10^4^ cells/well in the upper chamber of Matrigel-coated filters. Complete medium 600 μL was added to lower chamber as chemical attractant and cultured in an incubator at 37°C with 5% CO_2_. After incubation for 24 h, the cells that invaded to the bottom surface were fixed with 4% paraformaldehyde for 10 min, and then stained with 0.5% crystal violet for 15 min. Five visual fields were randomly observed with a microscope, and then photographed, counted, and statistically analyzed.

### Scratch wound healing assay

22RV1 cells were inoculated in 6-well plate at a density of 5 × 10^5^ cells/well with 2 ml cell suspension and cultured at 37°C until the cells grew to a monolayer covering the bottom of the culture plate. A scratch was made in the center of the monolayer using a 200 μL pipette tip. The cells were washed with PBS for three times and incubated with serum-free medium at 37°C with 5% CO_2_. The cells were observed and photographed at 0 h and 48 h.

### Cell apoptosis was detected by flow cytometry

Cell apoptosis was analyzed using apoptosis detection kit (A211-02, Vazyme, Nanjing, China). Firstly, 1 × 10^6^ cells were inoculated into 6-well plate and cultured at 37°C in 5% CO_2_ overnight (16–24 h). After cell collection, 100 μL binding buffer was added to re-suspended cells. Then 5 μL Annexin V-FITC and 5 μL Propidium Iodide were added to mix gently, and then incubated at room temperature for 15 min in the dark. The 400 μL binding buffer was added next, and the sample was mixed gently. The sample was detected by flow cytometry within 1 h.

### Animal study

A total of ten 4-week-old male nude BALB/c mice were purchased from the Institute of Comparative Medicine of Yangzhou University (Yangzhou, China). All animal studies were approved by the Animal Care Committee of General Hospital of Ningxia Medical University. The mice were randomly divided into two groups, including control group and hsa-mir-1247-transfected 22RV1 cell group (hsa-mir-1247-transfected group) (*n* = 5 each group). Then, 100 μL prepared cell suspension (1 × 10^7^/mL) was subcutaneously injected into the right axilla of nude mice. The tumor sizes were monitored every other day for 4 weeks. Finally, all mice were euthanized by cervical dislocation under anesthesia (pentobarbital sodium, 50 mg/kg) and the tumors were excised.

All data were presented as Mean ± SD and analyzed in GraphPad Prism 9. The difference between two groups was determined by *t* test, and *P* < 0.05 was considered statistical significance.

### Statistical analysis

Cell experimental data were expressed as mean ± standard error of the mean. Statistical differences between the two groups were calculated by Student’s *t*-test, and statistical differences between multiple groups were calculated by one-way ANOVA. R programming software was conducted on statistical analysis of bioinformatics data (version 4.0.3). *p* value < 0.05 was considered as statistically significant.

### Availability of data and materials

The data that support the findings of this study are openly available in The Cancer Genome Atlas database (TCGA, https://tcga-data.nci.nih.gov/tcga/).

## Supplementary Materials

Supplementary Table 1

Supplementary Table 2

Supplementary Table 3

Supplementary Table 4
